# Eslicarbazepine‐induced hyponatremia: A retrospective single‐center real clinical practice study

**DOI:** 10.1002/epi4.12814

**Published:** 2023-11-10

**Authors:** Ondřej Strýček, Vít Všianský, Irena Doležalová, Jitka Kočvarová, Martin Pail, Milan Brázdil

**Affiliations:** ^1^ Brno Epilepsy Center, First Department of Neurology, St. Anne's University Hospital and Faculty of Medicine Masaryk University, Member of ERN‐EpiCARE Brno Czech Republic; ^2^ Central European Institute of Technology (CEITEC) Masaryk University Brno Czech Republic

**Keywords:** eslicarbazepine, hyponatremia, risk factor

## Abstract

Hyponatremia is a typical side effect of antiseizure drugs from the dibenzazepine family. The study investigated the prevalence of hyponatremia in patients with epilepsy who were treated with eslicarbazepine. We aimed to determine the prevalence of hyponatremia, reveal the factors leading to the discontinuation of treatment, and identify possible risk factors for the development of hyponatremia including the dose dependency. The medical records of 164 patients with epilepsy taking eslicarbazepine in our center were analyzed. The overall prevalence of hyponatremia was 30.5%. The prevalence of mild hyponatremia, seen in 14%–20% of patients, was not dose dependent. The prevalence of moderate and severe hyponatremia was significantly dose dependent. The severity of hyponatremia was significantly dose dependent. Severe hyponatremia was found in 6.1% of patients. Hyponatremia was asymptomatic in the majority of cases, and in 48% did not require any management. Hyponatremia was the reason for discontinuation in 6.2% of patients. The major risk factor for developing hyponatremia was older age. The study shows that eslicarbazepine‐induced hyponatremia is usually mild and asymptomatic. It usually does not require any management and seldom leads to treatment discontinuation. Hyponatremia is dose dependent. Another major risk for developing hyponatremia (besides dose) is older age.

## 1 INTRODUCTION

Eslicarbazepine acetate (ESL) is an antiseizure medication (ASM) used for the treatment of focal‐onset seizures. ESL is a third‐generation ASM from the dibenzazepine family, which also includes carbamazepine (CBZ) and oxcarbazepine (OXC). Dibenzazepines are voltage‐gated sodium channel antagonists. All dibenzazepines cause hyponatremia, probably via stimulation of the vasopressin 2 receptor/aquaporin 2 pathway.^1^

The prevalence of hyponatremia (<135 mmol/L) induced by members of dibenzazepine family ASM differs across studies. Hyponatremia is reported in 12.5%–26% of CBZ‐treated patients and in 9.9%–46% of OXC‐treated patients.^1^, ^2^, ^3^ In clinical trials and open‐label studies, the prevalence of hyponatremia in ESL‐treated patients was under 6%.^4^, ^5^, ^6^ More recent studies showed a much higher prevalence of hyponatremia in one‐third of ESL‐treated patients.^7^ Compared to OXC, the prevalence of ESL‐induced hyponatremia is not statistically different (43% of OXC‐treated patients; 33% of ESL‐treated patients), but both are significantly higher than with CBZ (16% of CBZ‐treated patients).^7^ Results comparing the correlation between drug dose and hyponatremia severity are varied because the correlation is weak. CBZ‐induced hyponatremia and OXC‐induced hyponatremia are probably related to dosage.^1^, ^7^ In ESL‐related hyponatremia, reports are contradictory, showing dose dependence and independence.^5^, ^7^

ESL‐induced hyponatremia (and CBZ‐ and OXC‐induced hyponatremia) is usually mild or moderate, asymptomatic, and rarely leads to discontinuation of therapy.^4^, ^5^ Hyponatremia lower than 125 mmol/L is seen in 1.5% of patients, and hyponatremia under 120 mmol/L is extremely rare during ESL treatment.^5^, ^7^, ^8^

There are a few risk factors for dibenzazepine‐induced hyponatremia. For CBZ‐ and OXC‐related hyponatremia, these risk factors are older age and concomitant use of a high‐risk medication. Risk‐related medications are antihypertensive drugs, especially diuretics.^1^, ^7^, ^9^, ^10^ Older age is also a risk factor for ESL‐related hyponatremia; other factors have not been explored.^7^

There are only two real clinical practice studies (outside clinical trials) reporting ESL‐induced hyponatremia with a low number of patients,^1^, ^7^ and the findings of previous studies are contradictory. We performed a retrospective monocentric study to explore the prevalence of hyponatremia in patients treated for epilepsy, the types of management, and the possible risk factors.

## 2 METHODS

We analyzed the medical records of patients with epilepsy taking ESL in our outpatient clinic from 2010 to 2022. We compared sodium levels before ESL administration and levels during titration (at 400 mg, 800 mg, 1200 mg, and 1600 mg/day if applicable). ESL was titrated between 400 and 1200 mg once daily during at least a 1‐month observation period.

Hyponatremia was defined as a sodium level below 135 mmol/L (mild 130–134 mmol/L, moderate 125–129 mmol/L, and severe under 125 mmol/L).^11^ Following the results of earlier studies, age, gender, antidiuretic drugs, and other (than diuretic) drugs causing hyponatremia were counted as covariables related to hyponatremia.^1^, ^7^, ^9^, ^10^ Citalopram is a widely used antidepressant causing hyponatremia (with a reported 16.5% prevalence of hyponatremia after starting treatment); we particularly explored citalopram administration as a covariable related to hyponatremia.^12^

Only patients with known natremia before ESL administration were included in the study. Patients with hyponatremia before ESL administration were excluded, with the exception of patients with previously documented CBZ‐induced hyponatremia (switching from CBZ). Other exclusion criteria were pregnancy and other medical conditions causing hyponatremia. In patients with ESL‐induced hyponatremia, the type of management was analyzed. If ESL therapy was discontinued, the reason was evaluated. The clinician was free to decrease the dose or withdraw ESL in case of side effects.

A paired *t*‐test was used to compare the values of natremia before treatment with ESL and among various dosages. To determine the dependency of natremia on ESL dosage, one‐way repeated measures of analysis of variance (ANOVA) was used. The analysis of all risk factors for hyponatremia was performed using a logistic regression model with standard checks for multicollinearity. Two‐sample proportion tests were used to determine the differences in hyponatremia frequency among the various patient groups. The level of statistical significance was set at *α* ≤ 0.05. The R software package was employed to conduct the complete statistical analysis.

## 3 RESULTS

A total number of 164 patients receiving ESL were included in the study. Nine of the patients (5.5%) had hyponatremia before ESL administration. Hyponatremia was found in 50 patients (30.5%). Table 1 compares the prevalence of natremia in patients according to ESL daily dose. Mild hyponatremia was present in roughly the same proportion (14%–20%) in all the standard dose categories (from daily doses of 400 to 1200 mg), moderate and severe hyponatremia were found only in daily doses of 800 mg and higher. The prevalence of hyponatremia was higher in the group taking a daily dose of 1600 mg than in the group taking a daily dose of 1200 mg. The natremia level change increased significantly with increasing daily doses. In only one case (0.6%) was the hyponatremia symptomatic. Table 2 shows demographic data, reasons for ESL treatment discontinuation, hyponatremia management, and observed risk factors. The most common reason for discontinuation was treatment ineffectiveness (9.8%). Hyponatremia was the reason for discontinuation in 6.7% of subjects. Of the patients with ESL‐induced hyponatremia, 14.0% had previously documented CBZ‐induced hyponatremia. Almost half of the patients with hyponatremia did not require any management (48%); 24% of patients received sodium substitution and ESL was withdrawn in 22%. Regarding concurrent medication, hyponatremia was rare in patients taking citalopram, diuretics, or other drugs (less than 3.0%). Table 3 shows the comparison of patients who did and did not develop hyponatremia after ESL administration, revealing possible risk factors. Significant differences were found in the ages of patients. The median age of patients developing hyponatremia during ESL treatment was 51 years, which significantly differs from the age of patients without hyponatremia (40 years). The significance was more evident when focused particularly on older patients (age ≥65 years). Older patients are a notable portion (22%) of the patients with ESL‐induced hyponatremia; older patients are 7% of the normal natremia group. Other risk factors did not show significant differences in these patient groups. Twenty‐three patients switched from CBZ to ESL. Five of them had documented CBZ‐induced hyponatremia. These five patients all had hyponatremia during ESL treatment too; a significant number of patients (6, *P* < 0.001) newly developed hyponatremia. We did not find gender or taking citalopram as a risk factor. There were not enough patients for statistical analysis in groups of patients taking diuretics and other hyponatremia‐causing medication.

**TABLE 1:** Prevalence of eslicarbazepine dose‐dependent hyponatremia.

Daily dose	Normal natremia, n	Mild hyponatremia (130–134 mmol/L), n	Moderate hyponatremia (125–129 mmol/L), n	Severe hyponatremia (<125 mmol/L), n	Change in natremia from baseline (mmol/L, ±SD)	*P*
400 mg	4 (80%)	1 (20%)	0	0	+0.2 (±0.7)	0.799
800 mg	69 (80%)	12 (14%)	2 (2%)	3 (3%)	**−2.4 (±0.4)**	**<0.0001**
1200 mg	58 (62%)	18 (19%)	11 (12%)	6 (6%)	**−4.7 (±0.6)**	**<0.0001**
1600 mg	5 (71%)	0	1 (14%)	1 (14%)	**−5.7 (±2)**	**0.029**

*Note*: Significant changes in natremia are depicted in bold.

Abbreviations: mg, milligrams; mmol/L, millimoles per liter; n, number; *P*, *P*‐value; SD, standard deviation.

**TABLE 2:** Characteristics of patients.

Total number of patients	164	
Age in y, mean (SD)	45.4 (±14.3)	
Sex		
Male	93	56.7%
Female	71	43.3%
Reason for discontinuation, n (% from all)		
Not effective	16	9.8%
Other side effect	7	4.3%
Hyponatremia	11	6.7%
Hyponatremia management, n (% from patients with hyponatremia)		
Sodium substitution	12	24%
Discontinuation	11	22%
No change in management	27	54%
Hyponatremia risk factors, n (% from all)		
Switch from CBZ	23	14.0%
CBZ‐induced hyponatremia	5	3.0%
Citalopram	5	3.0%
Diuretic	2	1.2%
Other drugs causing hyponatremia	3	1.8%

Abbreviations: CBZ, carbamazepine; n, number; SD, standard deviation; y, years.

**TABLE 3:** A comparison of patients with hyponatremia and normal natremia during eslicarbazepine treatment.

	Hyponatremia	Normal natremia	*P*
Number	50	114	
Age y (median)	51	40	**0.00046**
Males/females, n	26/24	67/47	0.704
Risk factor			
Switch from CBZ, n (%)	11 (22%)	12 (10.5%)	0.948
Citalopram, n (%)	2 (4%)	3 (2.7%)	0.926
Diuretics, n (%)	1 (2%)	1 (1%)	X
Other drugs causing hyponatremia, n (%)	2 (4%)	1 (1%)	X
Age ≥65 years, n (%)	11 (22%)	7 (6%)	**0.00653**
No risk factor, n (%)	34 (68%)	97 (85%)	0.382

*Note*: Significant differences in proportion of patients in groups with hyponatremia and normal natremia during eslicarbazepine treatment are depicted in bold *P*‐value.

Abbreviations: CBZ, carbamazepine; n, number; *P*, *P*‐value; SD, standard deviation; y, years.

## 4 DISCUSSION

We studied ESL‐induced hyponatremia in patients with epilepsy. The overall prevalence of hyponatremia was 30.5%. In the majority of cases, the hyponatremia was mild. The prevalence was higher than in randomized clinical trials (RCT) but comparable with real clinical practice studies, reporting hyponatremia in almost one‐third of the patients, which probably more accurately reflects reality without any preselection of patients.^4^, ^5^, ^6^, ^7^ The prevalence of mild hyponatremia was not dose dependent and was seen in 14%–20% of patients. The prevalence of moderate and severe hyponatremia was significantly dose dependent.

The severity of hyponatremia was also significantly dose dependent. Moderate and severe hyponatremia was found only in daily doses of 800 mg and higher. With increasing daily ESL doses (up to 1600 mg daily), there was a significant change in natremia from the baseline. Previous results comprising the correlation between drug dose and severity of hyponatremia were discordant because the correlation was reported as weak.^5^, ^7^ In our study, we show that ESL‐related hyponatremia is dose related, up to a daily dose of 1600 mg. Severe hyponatremia was found in 6.1% of patients, which is higher than the previously reported prevalence of 1.5% of patients.^5^

The most common reason for discontinuation was drug treatment ineffectiveness (12.9%). Hyponatremia was the reason for discontinuation in 6.2% of all patients; it was the reason for discontinuation in 22% of the patients with hyponatremia. Hyponatremia was symptomatic in 0.5% of the patients, which corresponds with the very low overall prevalence of symptomatic ESL‐induced hyponatremia seen in RCT studies.^4^, ^5^ The reasons for discontinuation of ESL in cases of asymptomatic hyponatremia were patient concerns about the treatment leading to possible worsening of sodium levels, even though they had no problems. In almost half of all patients, hyponatremia did not require any treatment (hyponatremia was mild and asymptomatic); 24% of patients needed a sodium substitution.

We confirm the findings of previous studies that age is a major significant factor for developing ESL‐induced hyponatremia.^7^ We also show that older people in particular make up a significant portion of patients with hyponatremia during ESL treatment. These findings indicate higher caution for possible hyponatremia (and more regular sodium level controls) when starting ESL treatment in older patients. Other than age, we did not identify other risk factors such as concomitant medication or gender.

Among patients switching to ESL treatment from CBZ, all the patients with CBZ‐induced hyponatremia also had hyponatremia during the ESL treatment. Additionally, a significant number newly developed hyponatremia during ESL treatment. This indicates a need for attention to sodium levels in patients starting ESL treatment, even if they had normal natremia during CBZ therapy. Other risk factors such as gender, concurrent medication of citalopram, diuretics, and other drugs causing hyponatremia did not lead to a significantly higher risk of hyponatremia, although there could be bias due to the low number of patients.

## 5 CONCLUSION

Hyponatremia is relatively common among ESL‐treated patients: It is found in almost one‐third of cases (30.5%). ESL‐induced hyponatremia is usually mild; severe hyponatremia is exceptional. The risk of hyponatremia increases with higher doses, with a maximum change of natremia in doses up to 1600 mg per day. Hyponatremia is typically asymptomatic, requires no management, and rarely leads to discontinuation of treatment. A major risk for developing hyponatremia is older age; people over 65 years old are particularly vulnerable. Normal natremia during CBZ treatment does not predict normal sodium levels during ESL therapy.

## AUTHOR CONTRIBUTIONS

Ondřej Strýček was involved in literature analysis, study design, data acquisition, writing and approval of the manuscript. Vít Všianský was involved in data acquisition, data analysis, writing and approval of the manuscript. Irena Doležalová, Jitka Kočvarová, and Martin Pail were involved in data acquisition and approval of the manuscript. Milan Brázdil was involved in data acquisition, review and critique and approval of the manuscript.

## FUNDING INFORMATION

Supported by project nr. LX22NPO5107 (MEYS): Financed by European Union – Next Generation EU.

## CONFLICT OF INTEREST STATEMENT

None of the authors has any conflict of interest to disclose.

## ETHICS STATEMENT

We confirm that we have read the Journal's position on issues involved in ethical publication and affirm that this report is consistent with those guidelines.
